# Survival Prediction for Postoperative Patients With Kidney Cancer Based on Computed Tomography Radiomics: Retrospective Cohort Study

**DOI:** 10.2196/73162

**Published:** 2025-10-21

**Authors:** Guizhen He, Liwen Lai, Guoan Yu, Haowen Pang, Xiaohui Yu, Huaiwen Zhang, Qingxiu Zhu, Yingzi Lv

**Affiliations:** 1 Department of Nephrology, The Third People's Hospital of Jingdezhen, 76 Taoyang Road, Xinchang, East Suburb, Zhushan District, Jingdezhen, 333000, China, 86 18779803522, 86 0798-8410812; 2 Department of Nephrology, The Third People's Hospital of Jingdezhen affiliated to Nanchang Medical College, Jingdezhen, China; 3 Department of Oncology, The Affiliated Hospital of Southwest Medical University, Luzhou, China; 4 Jiangxi Cancer Hospital & Institute, Jiangxi Clinical Research Center for Cancer, The Second Affiliated Hospital of Nanchang Medical College, Nanchang, 330029, China

**Keywords:** computed tomography radiomics, kidney cancer, postoperative, predictive survival, nomogram

## Abstract

**Background:**

Kidney cancer remains a significant challenge in oncology, with accurate prognostic assessment being crucial for postoperative management. While radiomics has shown promise in cancer prognosis, there is limited research on comprehensive models that effectively integrate radiomic features with clinical parameters for kidney cancer survival prediction.

**Objective:**

This study aimed to develop and validate a comprehensive computed tomography (CT) radiomics-based nomogram for predicting overall survival in postoperative patients with kidney cancer by integrating radiomic features with clinical parameters.

**Methods:**

Radiomic features were extracted from regions of interest in CT images of 207 postoperative patients with kidney cancer. The eigenvalue data of all radiomic features were processed using *z* score standardization and the R software package *GLMNet*. We integrated survival time, survival status, and radiomic features and screened these features using the least absolute shrinkage and selection operator–Cox regression method. We conducted 10-fold cross-validation to obtain an optimal model of 5 radiomic features. Multivariate Cox regression hazard models were established to analyze patients’ overall survival. The predictive ability of the nomogram (receiver operating characteristic curve and calibration curve) was evaluated using bootstrap resampling validation. Patients were divided into high- and low-risk groups based on the radiomic risk score cutoff value, and the Kaplan-Meier method was conducted to identify established models’ forecasting ability. Five radiomic features were screened for predictive model construction.

**Results:**

This retrospective analysis was conducted from April 2024 to July 2024 using data from The Cancer Imaging Archive public database. The final cohort included 207 patients (3 excluded from the initial 210) who underwent nephrectomy for kidney cancer. The median follow-up time was 33 (IQR 11-47) months. The receiver operating characteristic curve and area under the curve showed that the predictive model performed well. The calibration curve of nomogram and radiomic features in the cohort study set indicated an overall net benefit. Kaplan-Meier curves indicated that overall survival time was dramatically shorter in the high-risk group.

**Conclusions:**

Our radiomics nomogram successfully integrates CT-derived radiomic features with clinical variables for kidney cancer survival prediction, demonstrating good prognostic capability and offering a noninvasive, quantitative tool for personalized postoperative management and clinical decision-making.

## Introduction

### Background

Kidney cancer is the most common malignant cancer of the urinary system, and nephrectomy is the standard treatment for locoregional kidney cancer [1]. Progress has been achieved through multiple optional methods of surgical resection and systemic therapies for kidney cancer [2]. However, overall survival (OS) and prognosis, especially if the cancer is detected at an advanced stage, remain unsatisfactory if the cancer is not treated optimally because of its high invasiveness, high mortality, and resistance to chemoradiotherapy [2,3]. Furthermore, the incidence of kidney cancer has been reported to have steadily increased in recent years [4-6]. The ability to predict the prognosis preoperatively and noninvasively is vital; however, patients who undergo nephrectomy still lack specific radiomic markers because of the complexity of disease progression and high heterogeneity. Radiomic markers that can facilitate predicting and monitoring the prognosis with good accuracy are urgently needed, after which a personalized strategy for clinical treatment needs to be provided. However, there remains limited research on comprehensive prognostic models that effectively integrate radiomic features with clinical parameters for kidney cancer survival prediction [7].

Radiomics is a rapidly developing field in which medical images are transformed into available radiological data and allows for efficacy monitoring, prognosis surveillance, microenvironment evaluation, and biological behavior assessment through quantitatively extracting features and facilitating in-depth characterization of tumor phenotypes beyond imaging interpretation [8-10]. Clinically, the postoperative prognosis of patients with cancer is frequently evaluated using the tumor-node-metastasis (TNM) classification system [11]. Recently, there has been an increasing focus on the application of computed tomography (CT) radiomics in kidney cancer, which has satisfactory potential in terms of lesion characterization, histological grade, and assessment of response to treatment [12-14]. Nevertheless, the correlation between radiomic features and the prognosis of patients with kidney cancer remains unclear, and further research is required to provide references for the clinical setting.

### Objectives

To address this need, we developed a comprehensive radiomics-based nomogram that combines CT-derived features with clinical parameters for survival prediction in postoperative patients with kidney cancer. This integrated approach provides a practical tool for prognostic assessment and clinical decision-making.

## Methods

### Patient Selection and Clinical Data Collection

Data from 210 patients with kidney cancer were obtained from The Cancer Imaging Archive (TCIA) database [15]. Inclusion criteria were (1) pathologically confirmed kidney cancer, (2) having undergone nephrectomy, (3) available contrast-enhanced CT imaging data, and (4) complete survival follow-up data. Exclusion criteria were patients with an OS of 0 days (n=3). Our final cohort comprised 207 patients. Clinical characteristics collected included demographic data (age and gender), smoking history, comorbidities, tumor characteristics (pathology TNM stage, tumor histologic subtype, and tumor International Society of Urological Pathology grade), surgical parameters, and survival outcomes (vital status and survival time). To identify independent risk factors of OS, univariate and multivariate Cox regression analyses were conducted using the *survival* and *forest plot* packages in R (R Foundation for Statistical Computing).

### Radiomic Datasets and Feature Extraction

#### Image Preprocessing

CT images were preprocessed using wavelet-based methods. Before feature extraction, all images were resampled according to a voxel size of 1 × 1 × 1 mm^3^ to ensure standardized spatial resolution across the dataset.

#### Region of Interest Definition

Presegmented regions of interest, including gross tumor volume (GTV) and healthy kidney regions, were obtained from the TCIA database annotations. All regions of interest were visually inspected for quality and anatomical accuracy to ensure appropriate boundaries for radiomic analysis.

#### Feature Extraction

Feature extraction was based on the 3D Slicer platform and was conducted using the PyRadiomics package [16]. A total of 851 radiomic features were extracted from the GTV and healthy kidney regions, including shape features (volume, surface area, and sphericity), first-order statistical features (mean, variance, skewness, and kurtosis), and texture features (gray-level dependence matrix [GLDM], gray-level co-occurrence matrix, neighboring gray tone difference matrix, gray-level size zone matrix, and gray level run length matrix). Wavelet-transformed features from different frequency decompositions were also extracted. The eigenvalue data of all radiomic features were processed using *z* score standardization to ensure comparability across different feature scales [17].

### Feature Selection and Model Development

#### Feature Selection Rationale and Methodology

Least absolute shrinkage and selection operator (LASSO) regression was chosen for feature selection due to several advantages: (1) it effectively handles high-dimensional data with small sample sizes, which is common in radiomics studies; (2) it performs automatic feature selection by shrinking coefficients of less important features to 0; (3) it reduces overfitting risk through L1 regularization; and (4) it has been widely validated in survival analysis and radiomics research.

LASSO-Cox regression analysis was conducted using the *glmnet* package in R. We integrated survival time, survival status, and radiomic features from all 207 patients. The optimal regularization parameter (λ) was determined through 10-fold cross-validation using the minimum cross-validated partial likelihood deviance as the selection criterion. Features with nonzero coefficients at the optimal λ were selected for model construction.

#### Risk Score Construction and Stratification

A radiomic risk score was constructed using the linear combination of selected features weighted by their LASSO regression coefficients. The optimal cutoff value for risk stratification was determined using time-dependent receiver operating characteristic (ROC) curve analysis. Patients were then classified into high- and low-risk groups using this cutoff value.

### Statistical Analysis and Model Validation

#### Model Development and Nomogram Construction

Univariate and multivariate Cox regression analyses were conducted on the entire cohort to identify independent prognostic factors using the *survival* and *forestplot* packages in R. Clinical variables with *P*<.10 in univariate analysis were included in multivariate analysis along with the radiomic risk score. A nomogram was constructed using the *rms* package incorporating significant variables from multivariate analysis.

#### Model Performance Evaluation

Model performance was assessed using multiple metrics following established methodologies for machine learning–based cancer prediction studies [18]: discrimination (time-dependent ROC curves and area under the curve [AUC] at 1, 2, 4, and 6 years using the *survivalROC* package), calibration (calibration plots comparing predicted vs observed survival probabilities), clinical utility (decision curve analysis to evaluate clinical net benefit), and risk stratification (Kaplan-Meier survival curves with the log rank test to compare survival differences between risk groups) [19,20].

#### Validation Strategy

Given the presegmented nature of the TCIA dataset, we used bootstrap resampling for model validation. The 10-fold cross-validation was specifically used during the LASSO regression phase to determine the optimal regularization parameter (λ) for feature selection. For final model validation, we performed 1000 bootstrap iterations on the entire cohort (N=207) to assess model stability and obtain CIs for performance metrics.

#### Statistical Software and Significance

All analyses were conducted using the R software (version 3.6.3). A 2-sided *P*<.05 was considered statistically significant. Missing data were handled using complete case analysis.

### Ethical Considerations

This study was conducted in accordance with the Declaration of Helsinki and approved by the Ethics Committee of Jiangxi Cancer Hospital & Institute (2024ky057). As this study used publicly available databases, all data in this study have been anonymized or de-identified, so written informed consent from patients was not obtained.

## Results

### Clinical Data

This retrospective analysis was conducted from April 2024 to July 2024 using data from the TCIA public database. The final cohort included 207 patients (3 excluded from the initial 210) who underwent nephrectomy for kidney cancer. This study comprised 58.5% (121/207) male and 41.5% (86/207) female patients. The median follow-up time was 33 (range 1‐102; IQR 11-47) months at the time of analysis.

### Radiomic Feature Variable Selection

**Figure 1.**
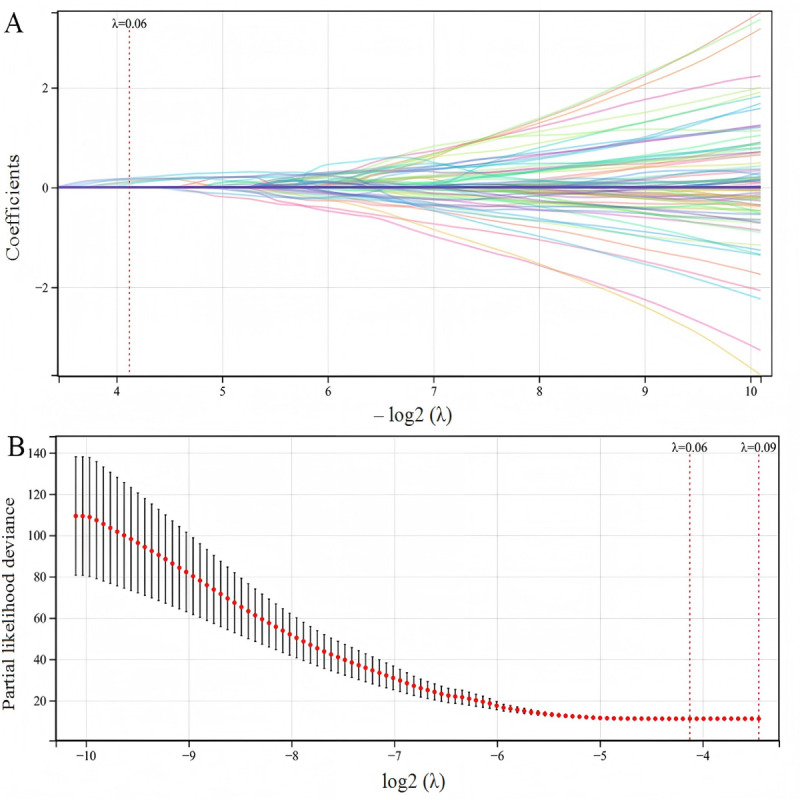
LASSO regression feature selection for kidney cancer survival prediction (entire cohort: N=207). (A) LASSO coefficient paths and (B) 10-fold cross-validation curve for selecting five radiomic features. LASSO: least absolute shrinkage and selection operator.

Five radiomic features were selected through LASSO-Cox regression analysis for OS prediction (Figure 1). The radiomic features included the following: kurtosis of first-order wavelet-LLH (H: high-frequency band; L: low-frequency band; GTV; feature 1), large-area high gray-level emphasis of the gray-level size zone matrix of wavelet-LHL (GTV; feature 2), dependence nonuniformity of GLDM of wavelet-HHL (healthy kidney; feature 3), long-run low gray-level emphasis of gray-level run length matrix of wavelet-LHL (GTV; feature 4), and large-dependence low gray-level emphasis of GLDM of wavelet-LLL (GTV; feature 5). The 5 selected radiomic features represent different aspects of tumor and tissue characteristics that may have clinical relevance. Texture features such as gray-level emphasis and dependence measures reflect tissue heterogeneity and organizational patterns, which may correlate with tumor biological behavior and aggressiveness. The inclusion of features from both tumor regions (GTV) and healthy kidney tissue suggests that both local tumor characteristics and the impact on surrounding normal tissue contribute to prognostic assessment. These features collectively capture quantitative imaging biomarkers that complement traditional clinical prognostic factors.

The variables from features 1 to 5 represent the significant radiomic features for predicting OS and are shown in Table 1. The formula for the OS radiomic risk score was as follows:


RiskScore=0.121279∗feature3+0.102978∗feature4+0.071174∗feature2+0.178144∗feature1+0.164676∗feature5

**Table 1.** Features 1 to 5, corresponding to different parameters for radiomics.

Feature number	CT^a^ radiomics
1	Kurtosis of first-order wavelet-LLH (GTV^b^)
2	Large-area high gray-level emphasis of GLSZM^c^ of wavelet-LHL (GTV)
3	Dependence nonuniformity of GLDM^d^ of wavelet-HHL (healthy kidney)
4	Long-run low gray-level emphasis of GLRLM^e^ of wavelet-LHL (GTV)
5	Large-dependence low gray-level emphasis of GLDM of wavelet-LLL (GTV)

a CT: computed tomography.

b GTV: gross tumor volume.

c GLSZM: gray-level size zone matrix.

d GLDM: gray-level dependence matrix.

e GLRLM: gray-level run length matrix.

### Establishment of Nomograms and Prediction Performance of the Nomogram Models

**Figure 2.**
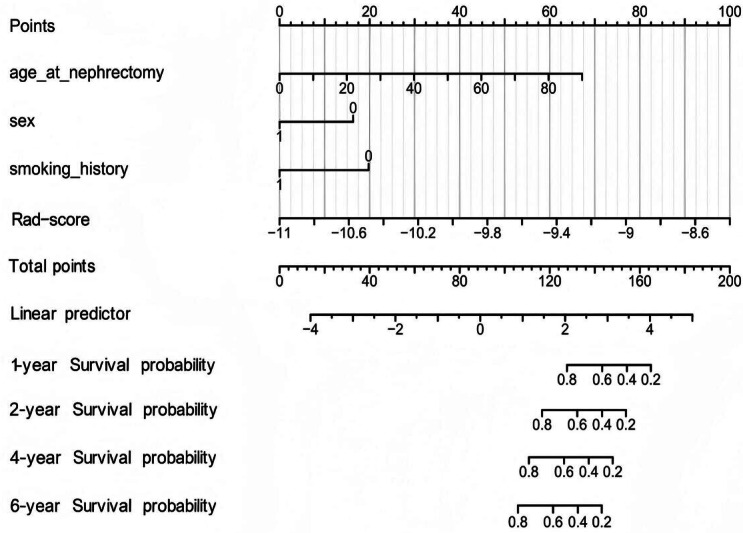
Nomogram prediction for 1-, 2-, 4-, and 6-year survival for patients with kidney cancer (entire cohort: N=207). Rad-score: radiomic risk score.

**Figure 3.**
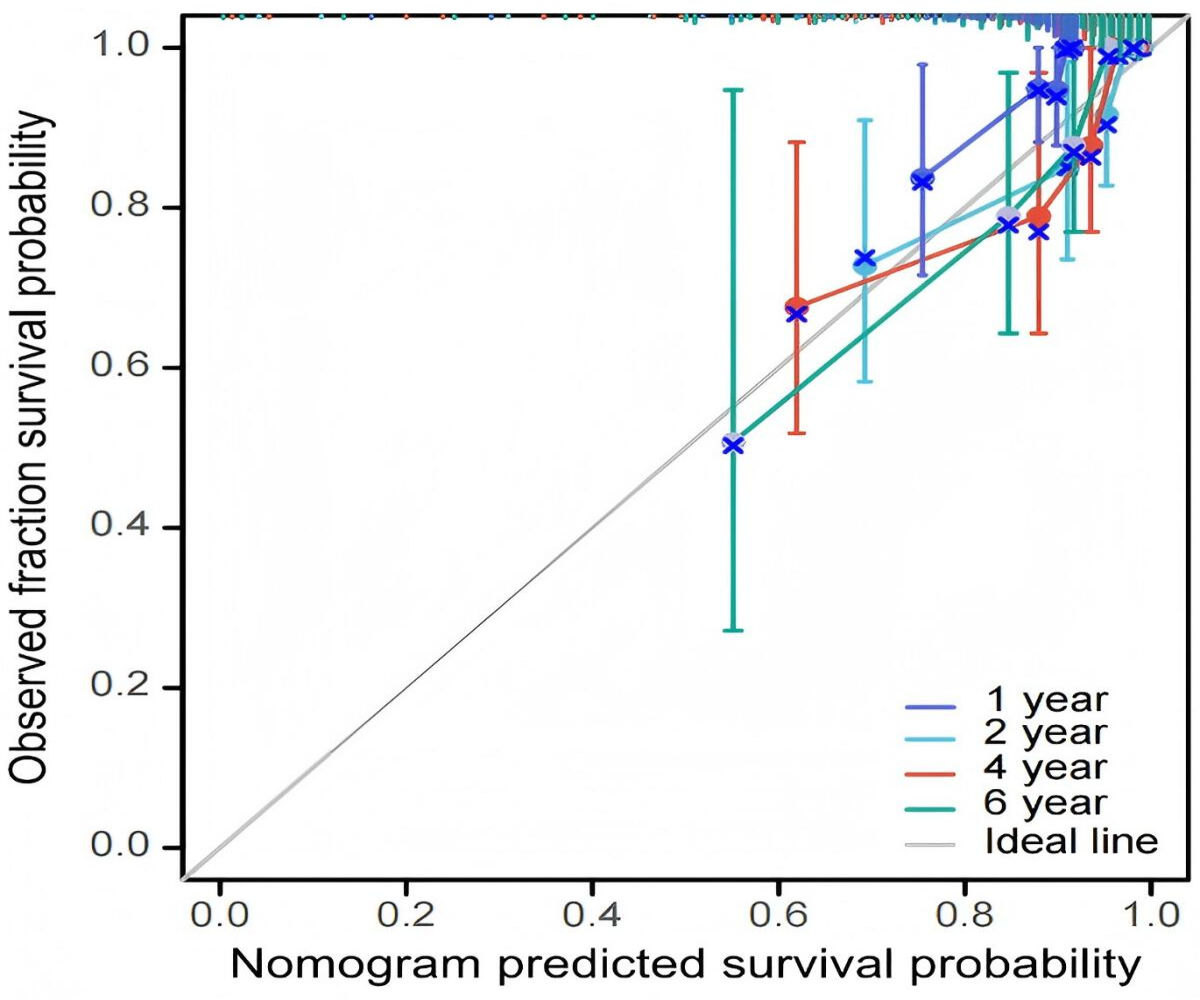
Kidney cancer overall survival nomogram calibration plots (entire cohort: N=207).

As shown in Figure 2, based on identified radiomic features, after integrating the data on survival time, survival status, and 5 features, we established a nomogram using the Cox method to assess the prognostic significance of these features in 207 patients. As shown in Figure 3, all calibration curves showed good results for the study samples.

### Model Validation Results

Bootstrap validation using 1000 iterations demonstrated robust model performance. The bootstrap-corrected AUC values were 0.80 (SD 0.03) for 1-year survival prediction, 0.77 (SD 0.04) for 2-year survival prediction, 0.74 (SD 0.04) for 4-year survival prediction, and 0.71 (SD 0.05) for 6-year survival prediction. The optimism-corrected concordance index was 0.73 (95% CI 0.68-0.78), indicating minimal overfitting.

### Association Between Radiomic Features and Survival Prognosis

**Figure 4.**
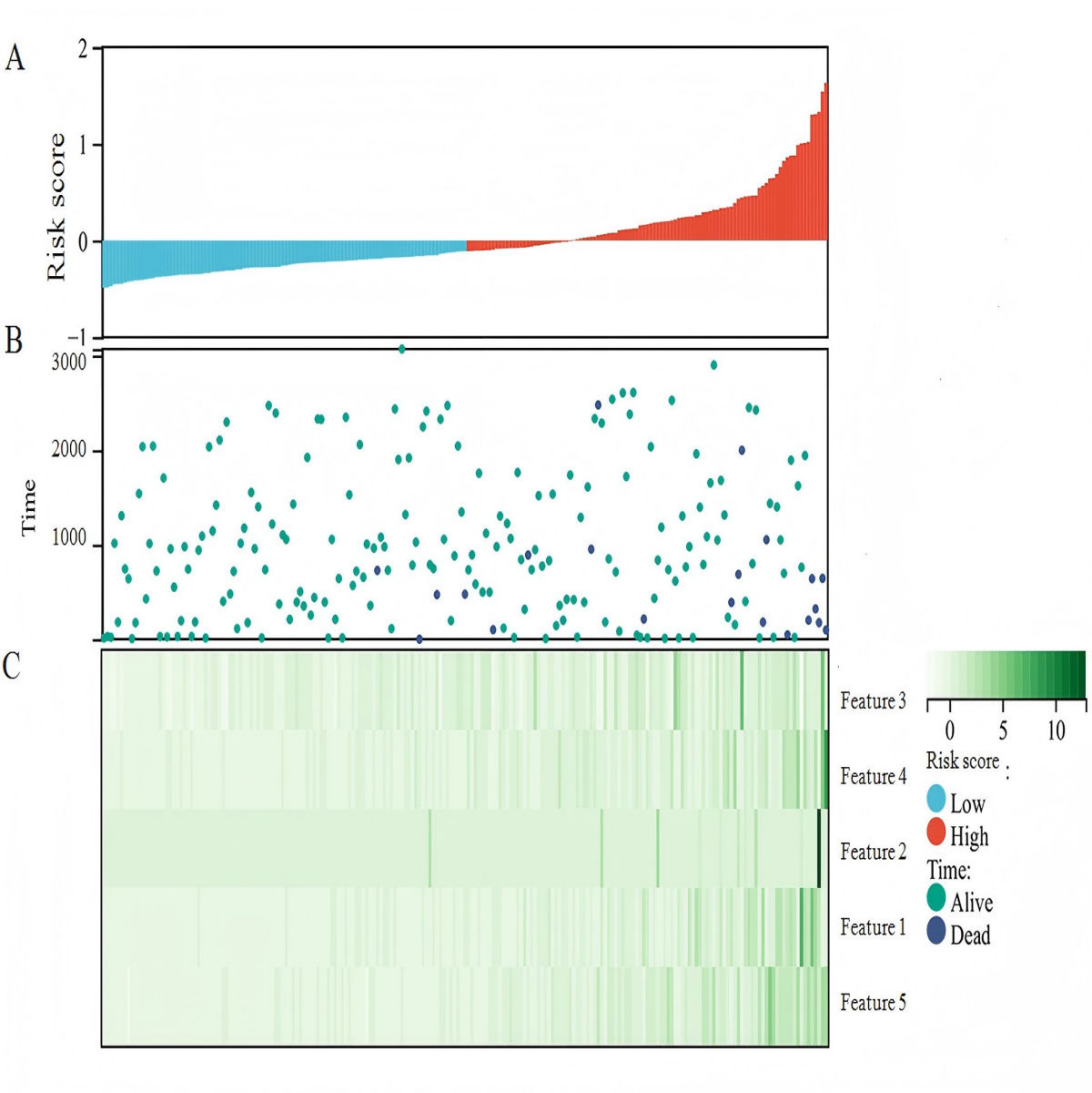
Risk stratification visualization showing the relationship between radiomic risk score and patient survival outcomes (entire cohort: N=207). The upper panel displays the distribution of risk scores ranked by patient survival time. The middle panel shows survival time for each patient. The lower panel illustrates the expression patterns of the 5 selected radiomic features (features 1-5) contributing to the risk score calculation. Color coding indicates patient survival status and risk group classification (high-risk vs low-risk groups based on the optimal cutoff value).

As shown in Figure 4, we analyzed the relationship between risk scores and patient outcomes across the entire cohort. The visualization clearly demonstrates that patients with higher risk scores experienced shorter survival times and higher mortality rates, as expected. The heat map pattern shows how the 5 radiomic features (features 1-5) collectively contribute to risk stratification, with distinct expression patterns between high-risk and low-risk patient groups. This risk score–based stratification effectively separates patients into clinically meaningful survival groups.

**Figure 5.**
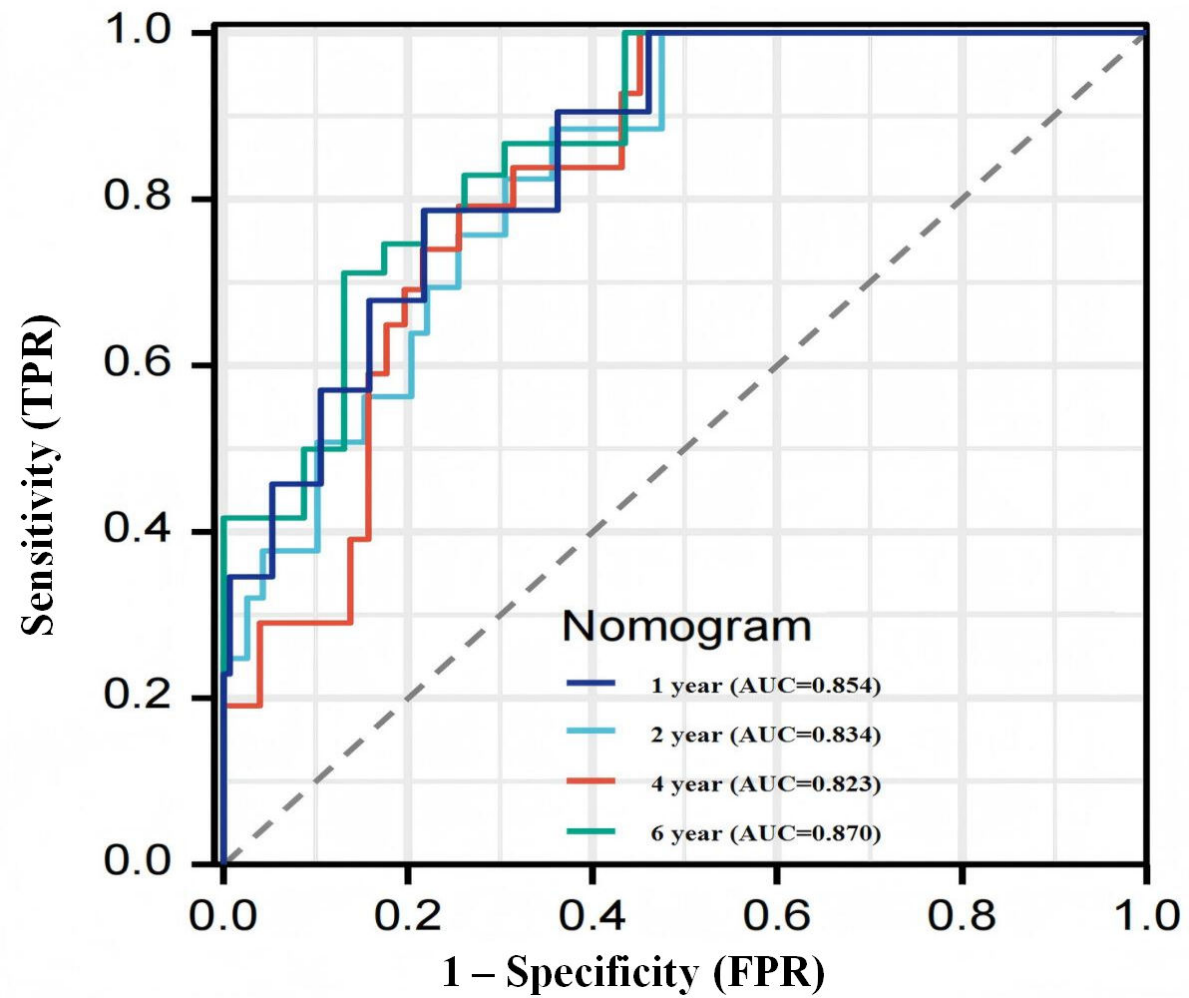
The 1-, 2-, 4-, and 6-year time-dependent receiver operating characteristic curves to assess the predictive accuracy for overall survival (entire cohort: N=207). AUC: area under the curve; FPR: false-positive rate; TPR: true-positive rate.

As shown in Figure 5, we used the time-varying ROC curve and AUC as the criteria for evaluating the nomogram. Figure 5 shows AUC of the model combining clinical features, and the radar score shows good performance.

### Kaplan-Meier Survival Analysis

**Figure 6.**
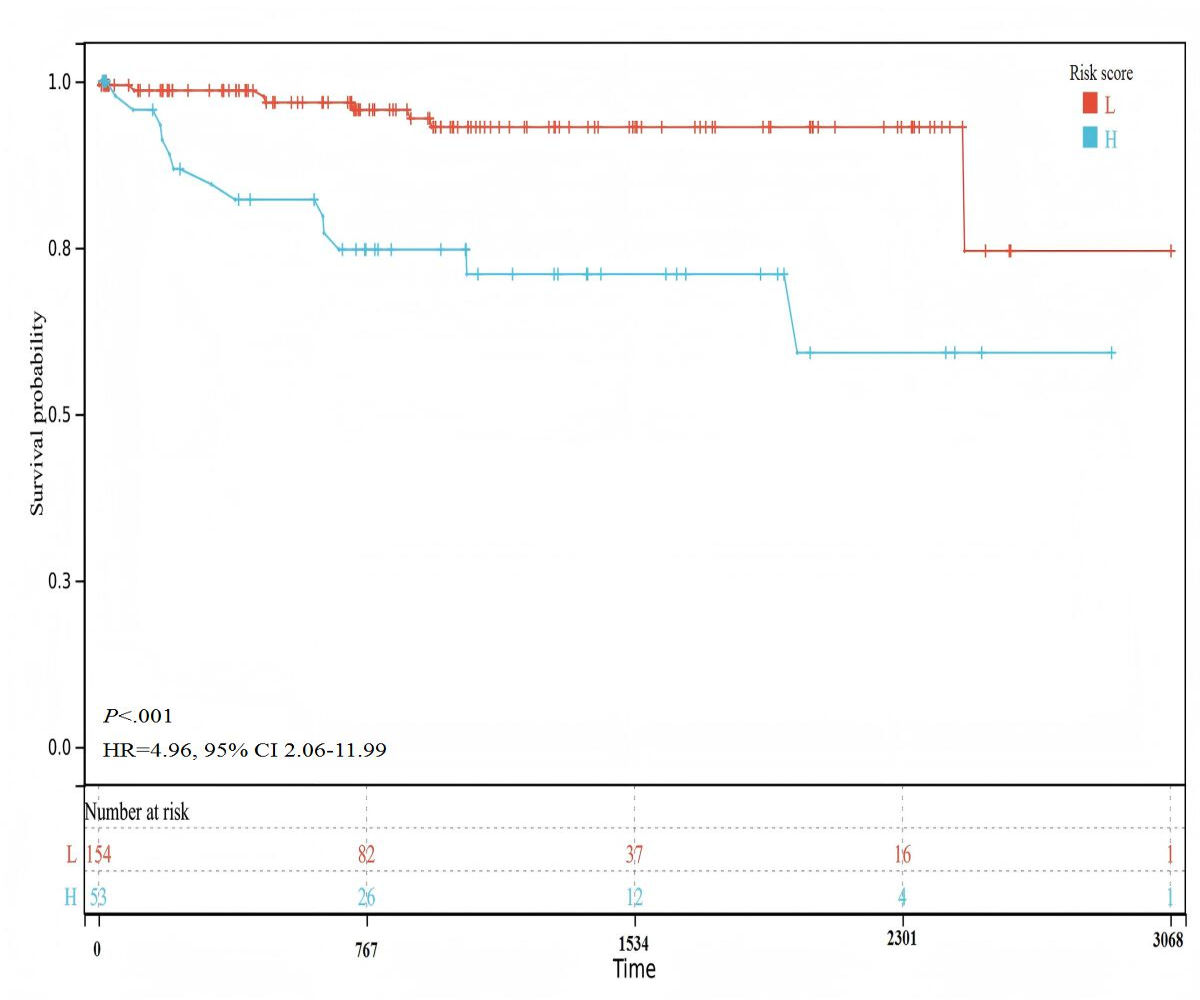
Survival curve depicting the high- and low-risk groups based on the radiomics score classification (entire cohort: N=207; low-risk group: n=154; high-risk group: n=53). H: high-risk group; HR: hazard ratio; L: low-risk group.

As shown in Figure 6, we divided the patients into low-risk and high-risk groups based on their radiomic characteristics. The cutoff value was 0.154921. The log rank test method was used to evaluate the significance of the prognostic difference between the groups, and a significant prognostic difference was observed (*P*<.001).

## Discussion

### Principal Findings

On the basis of publicly available data from the TCIA database, this study developed and validated prognostic nomogram models for patients with kidney cancer, and the results showed that our nomogram was advantageous for predicting patient prognosis. The nomogram could be useful for postoperative evaluation of patients with kidney cancer and development of individualized treatment plans. ROC and calibration curve results showed that the nomogram performed well in terms of noninvasive prediction of survival in patients with kidney cancer. The nomogram visually showed how to numerically quantify each factor for predicting survival in patients with kidney cancer. On the basis of our results, we observed good performance in terms of our radiomics characterization and the predict nomogram, suggesting that our model can be used to effectively predict the prognosis of patients with kidney cancer and create a robust decision-making framework for clinicians.

### Comparison With Previous Work

The incidence of kidney cancer has been steadily increasing over recent decades, although the reasons for this remain unclear [1]. The annual incidence of kidney cancer has been increasing in both men and women [21]. However, patients with the same type of kidney tumors might have different prognoses owing to the complex internal structure and high heterogeneity within tumors. Complete resection or percutaneous core histopathology biopsy remains a standard invasive method for assessing prognostic indicators (ie, histological classification, grades, and stages) of kidney cancer to guide further treatment [21,22]. An objective and noninvasive approach is needed to evaluate and predict the clinical outcomes of patients with kidney cancer. CT plays a vital role in the diagnosis and prognosis of renal disease as it is noninvasive and convenient, especially compared with biopsy, surgery, and immunohistochemistry. Biopsy is not always necessary because imaging is a highly accurate method for characterizing renal malignancy [23]. Radiomics, which is a commonly used method to extract characteristics in terms of mass data from each medical image, can provide tumor characteristics and functions at both macroscopic and micromolecular levels [24]. Recently, several studies have explored the biological progress of kidney cancer via the construction of radiomic models using CT images. Feng et al [25] and Kocak et al [26] showed that CT radiomics has the potential to predict the *BRCA1*-associated protein 1 mutation status in patients with kidney cancer. Ghosh et al [27] provided a radiomics-genetics pipeline that extracted 3D intratumor heterogeneity features from contrast-enhanced CT images and explored associations between features and gene mutation status. In 2001, a nomogram used to predict the 5-year survival of patients with kidney cancer was reported by Kattan et al [28]. The nomogram incorporated 4 factors: symptoms, histological subtype, tumor size, and TNM stage (1997 version) [28]. In 2018, Zhang et al [29] developed a nomogram specifically to assess the prognosis of patients with renal clear cell carcinoma postoperatively based on clinical data from 35,151 patients. However, several studies addressing the roles of immune profiles have encountered various uncertainties and have used constructed classification models to preoperatively identify pathological grades for patients with kidney cancer using machine learning–based CT radiomics with noninvasion [30-32]. Some studies have also shown the significance of CT radiomics in distinguishing kidney cancer from other renal mass diseases. Yang et al [33] developed various machine learning–based classification models to differentiate renal angiomyolipoma and kidney cancer, which performed well. Coy et al [34] reported the utility of machine learning in differentiating kidney cancer from oncocytoma on routine CT images using their models, which were able to accurately predict renal lesion histology on imaging. Meng et al [35] proposed a CT-based radiomic method to distinguish sarcoma from kidney cancer, with a good diagnostic performance. This is the first study to provide a predictive nomogram showing that radiomics can be used independently to predict prognosis in patients with kidney cancer. Our results indicated that our model could be a pivotal tool for the prognostic surveillance of kidney cancer.

### Clinical Implications

Risk stratification with specific risk scores using a radiomic signature was accurately performed, and the predictive nomogram, which comprehensively integrated radiomic and clinical signatures, could effectively predict outcomes for patients with kidney cancer and facilitate decision-making for clinicians. The noninvasive nature of this approach makes it particularly valuable for routine clinical application, potentially reducing the need for invasive procedures while providing accurate prognostic information [36]. In clinical practice, the radiomics nomogram could be integrated into routine postoperative imaging review to guide surveillance intensity and treatment decisions. High-risk patients might benefit from more frequent monitoring or earlier intervention discussions, whereas low-risk patients could have less intensive follow-up schedules. The scoring system could be incorporated into existing hospital information systems using automated feature extraction, requiring minimal workflow changes for clinical staff.

### Methodological Considerations

Our analytical approach evolved during the course of this study. Initially, we planned to use a traditional 70:30 train-test split for model development and validation. However, when we examined the TCIA dataset more carefully, we found that the presegmented images and the relatively small sample size (N=207) would result in insufficient power for robust model validation using a holdout approach, particularly for survival outcomes at multiple time points.

Therefore, we modified our approach to use the entire cohort (N=207) for model development, with bootstrap resampling for internal validation. This is a well-established alternative when sample sizes are limited, as recommended in radiomics guidelines [37]. Bootstrap validation provides robust performance estimates and CIs while maximizing the use of available data. Our results indicate acceptable model performance without significant overfitting.

This methodological adaptation underscores the importance of flexibility when working with real-world datasets and the need for transparent reporting of analytical decisions in radiomics research.

### Limitations

This study had several limitations that should be acknowledged. First, our conclusions were based on data obtained from public databases, which inevitably have limitations due to patient selection characteristics and data availability constraints. To mitigate this limitation, we applied rigorous inclusion and exclusion criteria and standardized image preprocessing protocols. However, this may still affect the generalizability of our results. Future studies should incorporate multi-institutional datasets with standardized imaging protocols to improve data quality and external validity. Second, our study sample size was relatively small (207 patients with kidney cancer), which was insufficient for robust model development and validation. This limitation may affect the performance and efficiency of the predictive signatures and limit the statistical power of our analysis. Future research should aim to include larger, multicenter prospective cohorts to validate this predictive model and improve its reliability. Third, TNM staging was not selected as a clinical feature related to OS in this study because patients with hepatocellular carcinoma with portal vein tumor thrombosis are classified as being in the late clinical stage, which makes it challenging to predict OS using clinical staging as all patients have similar staging information. This may limit the comprehensive evaluation of traditional prognostic factors. Future studies should explore methods to better integrate staging information with radiomic features. Fourth, our model only explored tumor regions using imaging. To our knowledge, peripheral tumors provide biological information for prognosis monitoring, but we did not investigate peritumoral regions that might contain valuable prognostic information. Future research should explore the integration of peritumoral radiomic features to enhance predictive performance. Fifth, the TCIA dataset may introduce selection bias as cases were contributed by specific institutions with potentially different patient populations and referral patterns. In addition, heterogeneous CT imaging protocols across contributing institutions (scanner types, reconstruction algorithms, and contrast protocols) present challenges for radiomic feature standardization and reproducibility. These factors limit the direct clinical applicability of our model, requiring validation and potential recalibration for different imaging protocols and patient populations before clinical implementation.

### Future Directions

To advance radiomics-based prognostic modeling in kidney cancer, several key research directions should be prioritized. First, multicenter prospective validation studies using standardized imaging protocols are essential to establish the generalizability and clinical applicability of radiomics models across different institutions and patient populations. These studies should include larger collaborative cohorts (>500 patients) to improve statistical power and model reliability. Second, radiomics research should expand beyond tumor regions to systematically investigate peritumoral features and explore integration with clinical biomarkers to enhance predictive performance. In addition, focused investigation of patient subgroups, particularly those with shorter survival periods who may benefit most from accurate prognostic prediction, represents an important research priority. Finally, the development of automated radiomics workflows and real-time clinical decision support systems will be crucial for translating research findings into routine clinical practice and facilitating widespread adoption of radiomics-based prognostic tools [38,39].

### Conclusions

We developed a triphasic contrast-enhanced CT–based radiomics nomogram that combined clinical factors and radiomic features. This nomogram demonstrated favorable predictive efficacy for differentiating kidney cancer postoperative outcomes. The model, combining CT-extracted radiomic features, showed good potential for evaluating OS in patients treated with nephrectomy and may facilitate clinical management and prognostic evaluation of postoperative patients with kidney cancer. As a noninvasive and quantitative method, the radiomics nomogram represents a promising tool for personalized medicine in kidney cancer management.

## Abbreviations

CT: computed tomography
GLDM: gray-level dependence matrix
GTV: gross tumor volume
LASSO: least absolute shrinkage and selection operator
OS: overall survival
ROC: receiver operating characteristic
TCIA: The Cancer Imaging Archive
TNM: tumor-node-metastasis
